# Prolonged B Cell Depletion With Rituximab is Effective in Treating Refractory Pulmonary Granulomatous Inflammation in Granulomatosis With Polyangiitis (GPA)

**DOI:** 10.1097/MD.0000000000000229

**Published:** 2014-12-12

**Authors:** Scott R. Henderson, Susan J. Copley, Charles D. Pusey, Philip W. Ind, Alan D. Salama

**Affiliations:** From the Imperial College Kidney & Transplant Institute, Hammersmith Hospital, London, UK (SRH, CDP); Centre for Nephrology, Division of Medicine, University College London, London, UK (SRH, ADS); Radiology Department, Hammersmith Hospital, London, UK (SJC); Department of Respiratory Medicine, Hammersmith Hospital, London, UK (PWI)

## Abstract

Pulmonary nodule formation is a frequent feature of granulomatosis with polyangiitis (GPA). Traditional induction therapy includes methotrexate or cyclophosphamide, however, pulmonary nodules generally respond slower than vasculitic components of disease. Efficacy of rituximab (RTX) solely for the treatment of pulmonary nodules has not been assessed. In this observational cohort study, we report patient outcomes with RTX in GPA patients with pulmonary nodules who failed to achieve remission following conventional immunosuppression. Patients (n = 5) with persistent pulmonary nodules were identified from our clinic database and retrospectively evaluated. Systemic manifestations, inflammatory markers, disease activity, concurrent immunosuppression, and absolute B cell numbers were recorded pre-RTX and at 6 monthly intervals following treatment. Chest radiographs at each time point were scored by an experienced radiologist, blinded to clinical details. Five patients with GPA and PR3-ANCA were evaluated (2 male, 3 female), mean age 34 (22–52) years. Pulmonary nodules (median 4, range 2–6), with or without cavitation were present in all patients. RTX induced initial B cell depletion (<5 cells/μL) in all patients but re-population was observed in 3 patients. Repeated RTX treatment in these 3 and persistent B cell depletion in the whole cohort was associated with further significant radiological improvement. Radiographic scoring at each time interval showed reduction in both number of nodules (*P* = <0.0001) and largest nodule diameter (*P* = <0.0001) in all patients for at least 18 months following B cell depletion. In summary, RTX therapy induces resolution of pulmonary granulomatous inflammation in GPA following prolonged B cell depletion.

## INTRODUCTION

Anti-neutrophil cytoplasm antibody (ANCA)-associated small vessel vasculitis is characterized by life-threatening inflammation of vascular beds, resulting in pulmonary haemorrhage, and rapidly progressive glomerulonephritis. Renal involvement signals the development of severe generalized disease, which untreated is associated with a mortality rate of over 85%.^1^ ANCA-associated vasculitis (AAV) encompasses the clinical syndromes of granulomatosis with polyangiitis (GPA, formerly Wegener's granulomatosis), microscopic polyangiitis (MPA), and eosinophilic granulomatosis with polyangiitis (EGPA).^2^ Most often, diagnosis is accompanied by circulating autoantibodies directed against either proteinase-3 (PR3-ANCA) or myeloperoxidase (MPO-ANCA), contained within azurophilic granules or secretory vesicles in neutrophils and monocytes, respectively.^3^ GPA and MPA are more common than EGPA, and the latter is regarded to differ in pathogenesis, extent of organ involvement, and frequency of ANCA detection.^3^

The presence of granulomatous inflammation of the upper and lower respiratory tract is the hallmark of GPA and a distinguishing feature from MPA on clinical presentation. Localized granulomatous inflammation of the lungs, eyes or ears, nose and throat (ENT) can progress to early systemic and generalized disease with disseminated vasculitis, but also poses significant disease burden to patients. Relapse rates reach up to 38% at 5 years in GPA, and PR3-ANCA, which is strongly associated with GPA, acts an independent predictor of relapse.^4^ However, there is a lack of evidence base guiding treatment of predominant granulomatous inflammation typically seen in localized and early systemic disease and, given the relapsing course of disease over time, much emphasis has been placed on identifying less toxic treatments associated with better patient tolerability and improved clinical efficacy.^5,6^

Recent trials have demonstrated equivalence of the monoclonal anti-CD20 antibody rituximab (RTX) with cyclophosphamide.^7,8^ However, suggestions of poorer outcomes for refractory granulomatous inflammation treated with RTX have previously been reported.^9^ In early, open-label prospective studies, a lack of efficacy of RTX on granulomatous disease manifestations and variability in induction of remission of refractory GPA was observed.^10,11^ Limited remission of pulmonary lesions was also noted following comparison of the effect of RTX on granulomatous and vasculitic manifestations in GPA, with most patients showing some sign of improvement although a proportion of patients did not respond to RTX at all.^12^ By contrast, we and others have described success of RTX therapy on retro-orbital,^13^ head, neck, and pulmonary manifestations.^14^ However, the effect of RTX on specific radiographic appearances of pulmonary granulomatous inflammation has not been assessed and no insight as to what predicts outcome in patients is established.

Here, we demonstrate the safe and effective resolution of pulmonary granulomata in an observational cohort study of patients with GPA resistant to conventional therapy who were treated with RTX, at a reduced dosing regimen compared to previous reports, and suggest that prolonged B cell depletion is required for effective nodule resolution.

## PATIENTS AND METHODS

Patients with early systemic GPA and persistent pulmonary granulomatous lesions who required additional therapy and were treated with RTX were identified from our multi-disciplinary vasculitis clinic and retrospectively evaluated. All investigators abided by the “Ethical Principles for Medical Research Involving Human Subjects” outlined in the Declaration of Helsinki, and adopted in October 2000 by the World Medical Association.

All patients fulfilled diagnostic criteria outlined by the Chapel Hill Consensus Conference and the American College of Rheumatology.^2,15^ Clinical characteristics, demographics, and disease duration were recorded. Detailed history of previous immunosuppressive treatment was established following review of clinical case records. RTX treatment was the same in all patients and included 1 g boluses administered on 2 occasions, 2 weeks apart. Repopulation of peripheral blood B cells was defined as >5 cells/μL following previous B cell depletion (<5 cells/μL). A further course of RTX was administered on an individual patient basis when clinical indication was supported by B cell re-population.

Disease manifestations, organ involvement, vasculitis damage index (VDI), and Birmingham vasculitis activity score (BVAS) were recorded for each patient. C-reactive protein (CRP), total white cell count (WCC), serum creatinine, and PR3-ANCA titres were recorded in all patients. In addition, serum immunoglobulins (IgG, IgA, and IgM) were documented. Lactate dehydrogenase (LDH) was not routinely measured during treatment.

Chest x-rays (CXR), performed at 6 monthly intervals throughout each patient's treatment course, were scored by an experienced radiologist blinded to clinical and radiological details. Radiographic scoring was performed up until 18 months (*Patients 2, 4, and 5*), 24 months (*Patient 3*), and 38 months (*Patient 1*). Extent of pulmonary manifestations examined included cavitating and non-cavitating pulmonary nodules, largest nodule diameter, presence of fibrosis, or consolidation, and presence of ancillary features such as pleural effusions.^16^ Analyses were performed using descriptive statistics and one-way repeated measures ANOVA using GraphPad Prism™.

## RESULTS

### Clinical Characteristics

We identified 5 patients (3 female, 2 male) with persistent pulmonary nodules that had not responded to conventional immunosuppression and were treated with RTX. Disease manifestations in all 5 patients were confined to the ENT, ocular, and pulmonary systems, representing early systemic disease.^17^ PR3-ANCA antibodies were present in all patients. Mean patient age was 34 years (range 22–52). Disease duration, previous and current immunosuppressants prior to administration of RTX are summarized in Table 1. In addition, baseline VDI, BVAS, CRP, creatinine, and total WCC are also shown in Table 1. Median follow-up reached 22 months (range 18–38). There were no infectious complications recorded in any patients during the follow-up period. RTX was well tolerated with no adverse reactions to drug infusion recorded. Hospitalization following therapy did not occur in any patient.

**TABLE 1:**
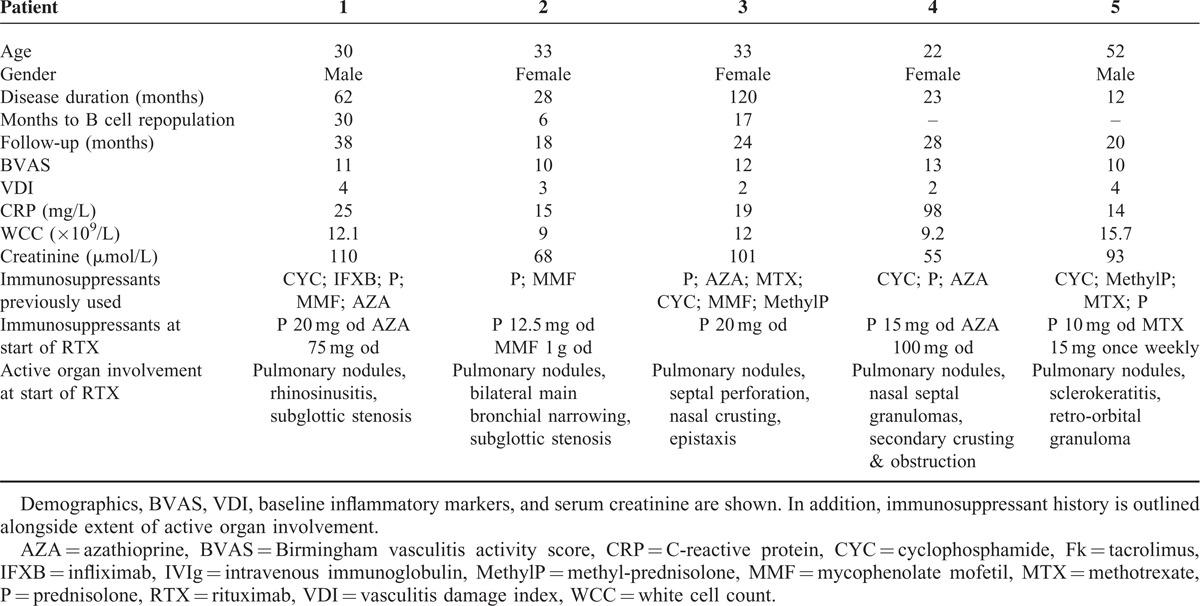
Clinical Characteristics of Patients Treated With RTX for Persistent Pulmonary Granulomatosis Inflammation in GPA

### Pulmonary Granulomata and Chest Radiography

**FIGURE 1:**
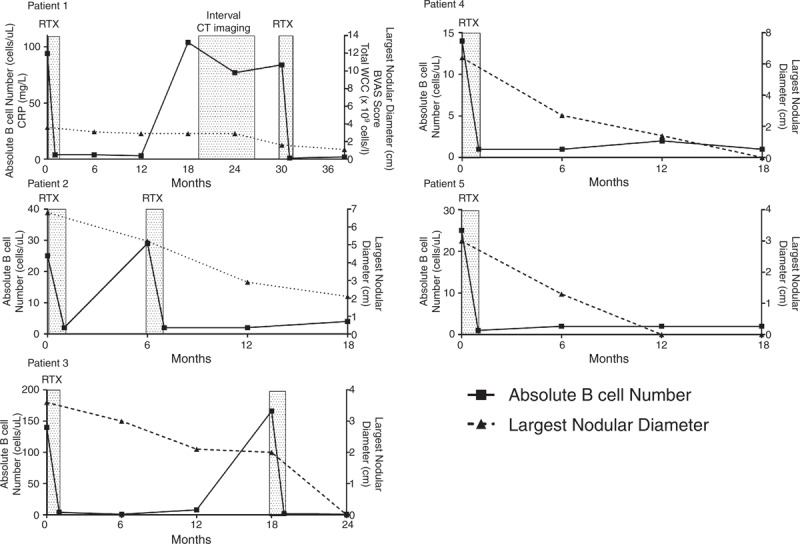
Change in peripheral blood B cell count after RTX (plotted against the left y axis) and size of largest pulmonary nodule diameter (plotted against the right *y* axis) is shown for each patient. RTX administration is represented by the shaded boxes and absolute peripheral B cell count 2 weeks after RTX therapy shows effective B cell depletion for each patient.

**FIGURE 2:**
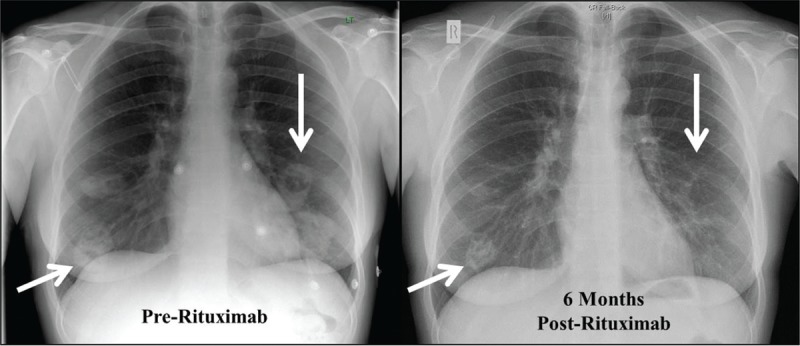
Chest radiographs performed pre-rituximab and after 6 months in Patient 2 are shown. Far left arrows show the largest cavitating pulmonary nodule, which reduced in size from 3.6 to 2.9 cm after treatment. The downward arrows to the right of each CXR show almost complete resolution of a cavitating nodule present at the start of treatment.

All patients had pulmonary nodules; cavitating (n = 2) and non-cavitating (n = 3). Nodules ranged in number, with a median of 4 nodules per patient (range 2–6), and in size, median 3.6 cm (largest diameter range 3–6.8 cm) prior to treatment. Change in largest diameter of pulmonary nodules in association with B cell depletion is shown in Figure 1. An example of a typical chest radiograph is shown in Figure 2. Arrows outline significant reduction in size and almost complete resolution of inflammation in some nodules. Consolidation (n = 1), fibrosis (n = 2), main bronchial stenosis (n = 1), and pleural effusions (n = 1) were additional features noted in some patients on initial chest radiology.

### Peripheral Blood B Cell Depletion, Disease Activity, and RTX Administration

Pre-treatment median BVAS score was 11 (10–13), VDI 3 (2–4), and mean CRP and WCC 34 (14–98) mg/L and 11.6 (9–15.7) × 10^9^ cells/L, respectively (Table 1). Peripheral blood B cell depletion (<5 cells/μL) was achieved in all patients by 2 weeks following RTX administration. B cells remained depleted at 6 months in 80% of patients. Further RTX was given to 3 patients (Patients 1–3) based on each individual's clinical condition, radiological changes, and B cell repopulation.

Patient 1 repopulated peripheral B cells at 18 months after initial RTX (104 cells/μL), however, clinically remained well, and B cell return was not associated with an inflammatory response (CRP 2 mg/L, WCC 7.7 × 10^9^ cells/L) or rise in BVAS (6 at 18 months, 13 pre-treatment). Therefore, the patient was monitored with interval imaging; at 18 months no change in size or number of pulmonary nodules was found (Figure 1), while high resolution CT (HRCT) performed at 24 months confirmed no change in pulmonary nodules and apparent reduction in mediastinal lymphadenopathy. The patient's condition also remained stable (CRP 2 mg/L, WCC 9.4 × 10^9^ cells/L and BVAS 6). PR3-ANCA antibody titres were variable throughout the treatment course but progressively increased to 420 units/mL at 30 months (*nadir* 76 units/mL at 12 months) in association with deterioration in clinical condition, with rise in BVAS to 11, rise in inflammatory markers (CRP 98 mg/L, WCC 12 × 10^9^ cells/L), sustained B cell repopulation (84 cells/μL), and persistent pulmonary nodules. Repeat RTX treatment was initiated at 30 months and the patient successfully depleted peripheral B cells within 2 weeks of administration.

B cell repopulation was observed at 6 months in Patient 2, despite achieving B cell depletion following initial treatment. Although a significant improvement in radiographic appearances was observed at 6 months (Figure 2), the clinical condition did not show the same response; BVAS was 10 at 6 months (13 pre-treatment), CRP 74 mg/L, and WCC 9.4 × 10^9^ cells/L. PR3-ANCA titres measured 69 units/mL pre-RTX and 40 units/mL at 6 months. With ongoing disease activity and signs of inflammation, further RTX was administered and the patient similarly depleted peripheral B cells within 2 weeks of administration, which was sustained up until the study end point at 18 months. This was associated with a sustained reduction of BVAS (to 1), CRP (to 5 mg/L), WCC (to 9.4 × 10^9^ cells/L), and pulmonary nodules.

After 12 months of therapy, B cell repopulation occurred in Patient 3 (8 cells/μL) and further increased at 18 months (166 cells/μL). BVAS was unchanged between 6 and 18 months (11 at 6 and 18 months, 12 pre-treatment), but WCC increased (12 × 10^9^ cells/L) and CXR at 18 months showed persistent pulmonary nodules with no evidence of infection. Therefore RTX was re-administered at 18 months and the patient depleted peripheral B cells as shown in Figure 1. CRP and PR3-ANCA titres showed minimal change between 6 and 18 months; 9 to 15 mg/L and 2 to 8 units/mL, respectively. BVAS reduced to 4 at 24 months.

In comparison, Patients 4 and 5 did not require any further RTX over an 18-month period with sustained B cell depletion after initial dosing. Prior to treatment, BVAS, CRP, and WCC measured 12, 98 mg/L and 9.2 × 10^9^ cells/L, respectively in Patient 4, and 16, 14 mg/L and 15.7 × 10^9^ cells/L, respectively in Patient 5. Resolution of inflammatory markers continued in both patients alongside B cell depletion. PR3-ANCA antibodies measured 64 units/mL at 18 months in Patient 4. Of note in Patient 5, PR3-ANCA antibody titre increased to 378 units/mL at 18 months in the absence of an inflammatory response, evidence of disease activity, or repopulation of peripheral B cells. Throughout treatment course, no patient developed any signs of renal involvement or intercurrent acute kidney injury and serum creatinine measured, at each clinic visit, remained within the normal reference range (data not shown).

### Outcomes

Prolonged B cell depletion (<5 cells/μL) following RTX was associated with significant reduction in number of pulmonary nodules (*P* = <0.0001) as well as size of the largest nodule (*P* = <0.0001) in all patients (pre-RTX to 18, 24, or 38 months post-RTX, respectively). BVAS reduced following treatment, as outlined for each patient above, however, “grumbling” ENT disease largely accounted for persistent disease activity. Pre-treatment median BVAS score was 11 (10–13) and post-treatment score was 4 (0–6), at 18 months (*Patients 2, 4, and 5*), 24 months (*Patient 3*), and 38 months (*Patient 1*), respectively. There was no overall significant difference in PR3-ANCA antibody concentration at 6 monthly intervals after RTX. Mean glucocorticoid dose pre-RTX was 12.5 mg (7.5–20 mg) which significantly decreased to a mean of 5 mg (5–7.5 mg) at 6 months (*P* = 0.008) and 2.5 mg (0–5 mg) at the study endpoint (*P* = <0.0001). Additional maintenance immunosuppression was not altered following RTX treatment.

Serial measurements of serum immunoglobulins were available in 4 patients (Patients 1–4). Mean serum IgG concentration measured 11.9 (9.9–14.6) g/L, 11.8 (8.7–13.2) g/L, 12.8 (7.8–18.3) g/L, and 11.5 (8.7–13.1) g/L at 0, 6, 12, and 18 months, respectively. Mean serum IgA concentration measured 2 (0.85–2.97) g/L, 1.8 (0.64–2.65) g/L, 1.7 (0.8–2.3) g/L, and 1.7 (0.8–2.7) g/L at 0, 6, 12, and 18 months, respectively. Mean IgM serum concentration measured 0.8 (0.1–1.5) g/L, 0.6 (0.2–1) g/L, 0.5 (0.2–0.8) g/L, and 0.4 (0.2–0.6) g/L at 0, 6, 12, and 18 months, respectively. Additional RTX treatment in Patients 1 and 3 after 18 months did not result in any further decrease in immunoglobulins.

## DISCUSSION

Although this represents a small retrospective (selected) series of patients, our data confirm that RTX is effective for treatment of pulmonary granulomas following a prolonged period of B cell depletion. Significant clinical improvement was delayed and took up to 2 years for complete radiological resolution of the nodules in some patients. BVAS score also improved after treatment, however, persistent ENT symptoms were scored as disease activity and it is worth considering the difficulty in clinically distinguishing between active disease and tissue damage and therefore BVAS may in fact over emphasize extent of disease activity after RTX. Nevertheless, our results demonstrate that in 5 patients with GPA, RTX improved pulmonary granulomatous manifestations when given in addition to standard therapy and allowed a significant reduction in corticosteroid usage.

RTX has been shown to be equally effective in treatment of ANCA negative GPA, suggesting that its effects are mediated in part via immune mechanisms distinct from autoantibody production. B cell depletion is therefore an attractive therapeutic strategy for treating granulomatous manifestations of GPA. Granulomata consist of neutrophils, giant cells, monocyte-derived tissue macrophages, B cells and CD4+CD28− T cells.^18^ The activation of T cells in an unbalanced Th1 phenotype and maturation of dendritic cells by PR3, exaggerating this Th1 response, have previously highlighted Th1 cells as critical mediators of granuloma pathogenesis in GPA.^18,19^ Indeed, both CD4+ Th1 and Th17 cells have been implicated as important orchestrators of granulomatous inflammation.

B cells also appear to play an important role in GPA granuloma pathogenesis. Significant B cell populations have been described within pulmonary granulomatous nodules and B cells have been shown to influence both monocyte and T cell activation. Whilst this study used peripheral blood B cell counts as an indicator of B cell repopulation, and this does not necessarily represent the proportion of B cells contained within pulmonary granulomata, it is becoming clearer that the balance of B cell subsets, particularly following RTX, is important in determining clinical outcome.^20^ Our group have characterized B cell subsets in AAV, by relative expression of CD24 and CD38. The balance of B cell subsets is altered after RTX and this altered phenotype is associated with decreased T cell activation and increased IL-10 production in vitro.^21^

It is therefore interesting to consider patients in whom B cells repopulated in the circulation, without supporting evidence of active inflammation. This was particularly apparent in Patient 1 where peripheral B cells repopulated but without a change in disease activity and radiological features showing stable and, to some extent resolving, inflammation. Evidence of worsening disease activity then developed 12 months later alongside sustained B cell repopulation and rising PR3-ANCA antibody titres. Phenotypic changes in repopulated B cells could account for this change in clinical course. This also emphasizes the additional benefit of RTX therapy in AAV, providing an opportunity to restore B cell homeostasis amidst an arsenal of autoantibody production against self-antigens and immune cell activation. In comparison, Patient 3 demonstrated significant PR3-ANCA antibody production without evidence of peripheral B cell repopulation, which emphasizes the need to consider granulomata in GPA as B cell containing tertiary lymphoid structures pivotal to driving the relapsing disease course.

Over the preceding decade, randomized controlled trials have standardized approaches to induction of remission and maintenance therapy.^6^ However, despite continuing improvement in patient outcomes with generalized disease, treatment of early systemic disease, and particularly granulomatous inflammation, confined to the respiratory and ENT tracts is not supported by the same evidence base to guide clinical practice.^22^ Disease relapse also remains common and PR3-ANCA antibody positivity, most often associated with GPA, and cardiac involvement are recognized as independent risk factors for relapse. In addition, serum creatinine shows a paradoxical inverse relationship with disease relapse; higher serum creatinine levels are associated with lower risk of relapse.^4^ Therefore, although localized and early systemic GPA lack organ or life-threatening manifestations, persistent granulomatous inflammation is difficult to treat and exposes patients to significant risks associated with long-term immunosuppression.^23^

The use of methotrexate, in combination with glucocorticoids, has been advocated following a randomized controlled trial, which demonstrated its equivalence at inducing remission in early systemic disease compared to oral cyclophosphamide. However, time to remission in patients with pulmonary involvement was increased, overall time to relapse was reduced and rate of relapse was greater in the methotrexate group, with notable adverse drug effects in each group.^24^ The beneficial effect of co-trimoxazole, alone or in combination with glucocorticoids, at achieving remission in localized GPA has been reported in single centre cohort studies.^25–27^ Similarly, rate of relapse remained high and additional immunosuppression was required in the majority of patients.^28,29^ Numerous biologic therapies have also been evaluated for refractory (most often defined as progressive disease unresponsive to conventional therapy, such as glucocorticoids and cyclophosphamide) and persistent disease.^6^ While cohort studies outline the use of deoxyspergualin, anti-thymocyte globulin, and TNF-alpha antagnosists, there is a greater level of evidence supporting the use of intravenous immunoglobulin and RTX for refractory and persistent disease.^1,30–34^

It is important to consider the impact of RTX on more than just B cell numbers. Whilst no apparent effect on immunoglobulins is observed after a single dose of RTX, IgM levels reduce over time and repeated dosing is associated with depletion of IgG levels and impaired humoral immune responses.^35–38^ Monitoring of serum immunoglobulin levels during prolonged treatment is therefore important.

In non-Hodgkin's lymphoma, from which the use of RTX originated, 4 RTX doses at 4 weekly intervals administered at 375 mg/m^2^ was shown to achieve peripheral B cell depletion for up to 6 months, after which gradual re-population of CD20-positive B cells was detectable. Clinical response was also shown to be influenced by serum antibody half-life, with values ranging from 12.7 to 370.8 hours following this standard infusion protocol.^39^ Previous reports in AAV have similarly employed 4 RTX doses at 4 weekly intervals administered at 375 mg/m^2^.^10–12^ However, in our series, we demonstrate that a standardized reduced dose of RTX (1 g boluses administered on 2 occasions, 2 weeks apart) at less frequent intervals, remains effective in maintaining B cell depletion with some patients not requiring any further B cell depletion therapy over an 18-month follow-up period. We therefore propose that maintaining depletion of B cells is a safer approach and guides individual patient response and improvement in pulmonary granulomatous manifestations in persistent disease, rather than total RTX dose administered.

There is no question that the efficacy of RTX on granulomatous lesions in GPA has not always been apparent,^9,11^ and it is clear that granulomatous features respond less rapidly than vasculitis features.^12^ Additionally, initial reports of resolution of granulomata with RTX were complicated by the co-adminstration of high-dose methylprednisolone.^12^ In our cohort, B cell depletion and improvement in granulomatous inflammation is unequivocally related to RTX, as methylprednisolone was not administered, concurrent immunosuppressive agents were not altered and we achieved a statistically significant reduction in prednisolone dosage following sustained B cell depletion.

Treatment of granulomatous lesions, in addition to the vasculitic component of GPA, is critical to inducing disease remission and minimizing patient morbidity, but is often harder to achieve and frequently entails the use of cyclophosphamide. RTX is now considered an effective treatment of refractory head and neck manifestations with remission rates reaching more than 80% in small cohort studies.^34^ This case series further develops the efficacy profile of RTX and supports cyclophosphamide avoidance in appropriate patients by providing a rationale for use of a reduced dosing regimen of RTX in persistent pulmonary granulomatous inflammation.
